# Patterns of long COVID symptoms among healthcare workers in the UK and variations by sociodemographic, clinical and occupational factors: a cross-sectional analysis of a nationwide study (UK-REACH)

**DOI:** 10.1177/01410768251389692

**Published:** 2025-12-08

**Authors:** Amani Al-Oraibi, Christopher A Martin, Katherine Woolf, Laura B Nellums, Carolyn Tarrant, Manish Pareek

**Affiliations:** 1 Department of Respiratory Sciences, University of Leicester, Leicester, UK; 2 Development Centre for Population Health, University of Leicester, Leicester, UK; 3 Centre For Academic Primary Care, School of Medicine, University of Nottingham, Nottingham, UK; 4 NIHR Leicester Biomedical Research Centre, Leicester, UK; 5 Department of Infection and HIV Medicine, University Hospitals of Leicester NHS Trust, Leicester, UK; 6 University College London Medical School, London, UK; 7 College of Population Health, University of New Mexico, Albuquerque, NM, USA; 8 Department of Population Health Sciences, University of Leicester, Leicester, UK; 9 NIHR Applied Research Collaboration East Midlands, Leicester, UK

**Keywords:** Long COVID, healthcare workers, symptoms, risk factors, cross-sectional, ethnicity

## Abstract

**Objectives:**

This study aimed to examine symptom patterns between healthcare workers (HCWs) with and without long COVID, identify the most common long COVID symptom groups and investigate how these symptom profiles vary across different ethnic groups, demographic characteristics, clinical factors and occupational roles in UK HCWS.

**Design:**

We conducted a cross-sectional study using data from the United Kingdom Research study into Ethnicity and COVID-19 outcomes in Healthcare workers (UK-REACH) cohort study. Data were collected electronically between October 2021 and October 2022.

**Setting:**

United Kingdom.

**Participants:**

Individuals aged 16 years or older, residing in the UK, working as HCWs or ancillary workers in a healthcare setting and/or registered with one of seven major UK healthcare professional regulators.

**Main outcome measures:**

Long COVID was defined as symptoms persisting for ⩾12 weeks following SARS-CoV-2 infection. Our primary outcome was the presence or absence of particular groups of long COVID symptoms. We collapsed 28 symptoms into seven groups: cardiopulmonary, gastrointestinal, musculoskeletal, neurocognitive/neurologic, upper respiratory tract, psychological/social and systemic.

**Results:**

Among 4033 HCWs with a history of COVID-19, those with long COVID (26.5%; 1067/4033) reported a higher prevalence of systemic, neurological and psychological symptoms compared with those without long COVID. Among those with long COVID, the most commonly reported symptom groups were neurocognitive/neurologic (63.4%), cardiopulmonary (40.0%) – highest among Asian HCWs at 45.6% – and systemic (54.6%), which particularly affected Black and Mixed ethnicities at 64.0% and 63.9%, respectively. In multivariable analyses, Asian HCWs had higher odds of experiencing cardiopulmonary symptoms (adjusted odds ratio (aOR): 1.62, 95% CI 1.04–2.51, *p* = 0.032), while female HCWs were more likely to experience gastrointestinal (aOR: 3.78, 95% CI 1.14–12.45, *p* = 0.029) and neurocognitive symptoms (aOR: 1.58, 95% CI 1.10–2.28, *p* = 0.014). Compared with those in medical roles, musculoskeletal symptoms were more commonly reported by those in nursing (aOR: 2.50, 95% CI 1.32-4.72, *p* = 0.005), allied health professional (aOR: 1.82, 95% CI 1.01–3.30, *p* = 0.048) and dental roles (aOR: 3.07, 95% CI 1.31–7.17, *p* = 0.010). Vaccination with two or three doses was protective against several symptom groups, including cardiopulmonary, musculoskeletal and neurocognitive symptoms.

**Conclusions:**

Our findings are the first to reveal distinct patterns in long COVID symptoms among HCWs with significant variations by ethnicity, sex and occupational role. These findings emphasise the need for targeted support strategies and workplace adjustments that consider both occupation-specific risks and individual sociodemographic factors.

## Introduction

As the coronavirus disease 2019 (COVID-19) pandemic progressed, it became increasingly apparent that many patients who recovered from acute SARS-CoV-2 infection were experiencing ongoing symptoms, which are now widely known as long COVID.^1^ Globally, it is estimated that 43% of COVID-19 patients report post-infection symptoms.^2^ Major health organisations, such as the National Institute for Health and Care Excellence (NICE) and the World Health Organization (WHO), have worked to identify the most common long COVID symptoms, which include fatigue, shortness of breath, cognitive issues, heart palpitations, headaches, diarrhoea and more.^3,4^ Over 200 different symptoms have been reported that impact everyday functioning. A wide range of systems can be affected – from respiratory and cardiovascular to neurological, gastrointestinal and psychological,^3,4^ but symptom profiles are still inconsistent and unclear.^3,4^

Healthcare workers (HCWs) are known to be at higher risk of SARS-CoV-2 infection than the general population; therefore, research on long COVID in this population is important. A cross-sectional study conducted in Latin America involving 2030 HCWs found that female HCWs, especially nurses, reported more symptoms than male physicians (median: 9 vs 6).^5^ A retrospective cohort study in Bangladesh estimated the frequency of long COVID symptoms among HCWs over time.^6^ Symptoms such as body aches, fatigue and neurological symptoms persisted in 5–8% of HCWs for up to a year, while 50% experienced loss of smell and taste, with 5–6% enduring these symptoms for over a year.^6^ A survey study in Iran involving 350 HCWs with a history of SARS-CoV-2 infection found that 75.7% of HCWs reported long COVID symptoms, with fatigue (53.1%), cough (43.1%) and muscle weakness (37.1%) being most common.^7^ These findings highlight the significant and diverse long-term symptoms experienced by HCWs following SARS-CoV-2 infection, with females and certain healthcare occupational roles demonstrating higher symptom burdens.

Researchers have emphasised the urgent need for data on the impact of long COVID among ethnic minorities and migrant populations, highlighting significant gaps in the current understanding of how these groups are affected.^8^ Comprehensive studies comparing long COVID symptom profiles among HCWs with different ethnic backgrounds in the UK are still lacking. Addressing this gap in the literature is crucial for developing targeted interventions and support for HCWs suffering from these symptoms.

We sought to address these knowledge gaps using data from the United Kingdom Research study into Ethnicity and COVID-19 outcomes in Healthcare workers (UK-REACH) longitudinal cohort study. Specifically, we aimed primarily to identify the most common long COVID symptom groups, examine how these symptom profiles vary across different ethnic groups and other demographic, clinical and occupational factors, and identify specific risk factors associated with different long COVID symptom groups among HCWs in the UK. Additionally, we aimed to examine differences in symptom patterns between HCWs with long COVID and those without long COVID.

## Methods

This study adhered to STROBE (Strengthening the Reporting of Observational Studies in Epidemiology) guidelines for reporting observational studies^9^ (see Supplemental Material 1).

### Overview

This analysis is based on data from the third (administered between October 2021 and November 2021) and fourth (administered between June 2022 and October 2022) questionnaires of the UK-REACH nationwide cohort study collected between October 2021 and October 2022. The study protocol,^10^ cohort profile^11^ and data dictionary (https://www.uk-reach.org/data-dictionary) provide detailed information on the study design, sampling and measures. We combined data from the third and fourth questionnaires to provide sufficient numbers for the analysis, where data from the third questionnaire were prioritised to reduce recall bias, as it was closer to the event of having COVID-19, ensuring more accurate reporting of symptom duration and severity; however, if missing, data from the fourth questionnaire were used where available. Combining data from both questionnaires was to enhance data completeness, as according to Little and Rubin, integrating data from multiple surveys is a good approach to handle missing data and enhance data completeness.^12^ This helped maintain the validity and reliability of our findings.^13^

### Study population

Participants included individuals aged 16 years or older, residing in the UK, working as HCWs or ancillary workers in a healthcare setting and/or registered with one of seven major UK healthcare professional regulators.^10^

### Cohort recruitment and formation of the analysis sample

**Figure 1.**
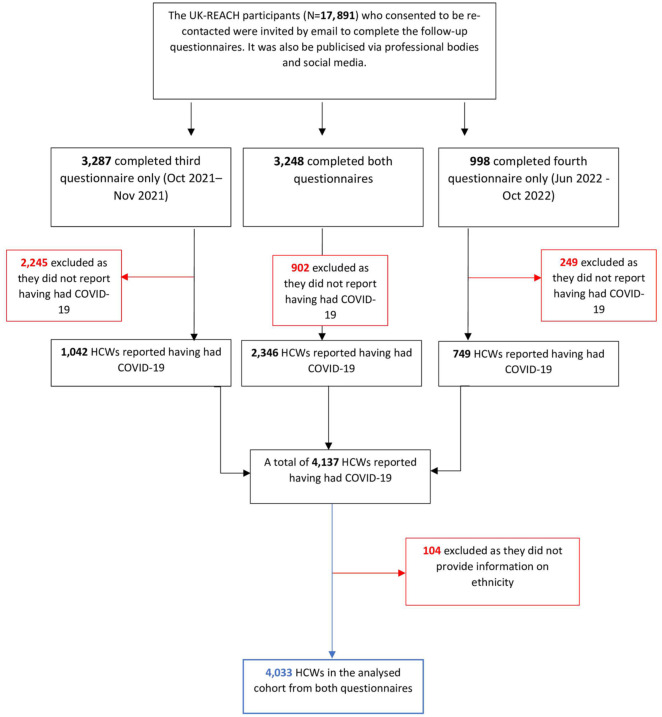
Formation of the analysed cohort. HCW: healthcare worker (those in professional healthcare roles or ancillary workers in a healthcare setting or registered with one of the seven participating UK healthcare professional regulatory bodies – see Methods for participating regulatory bodies).

Figure 1 illustrates the recruitment and formation of the analysed cohort. The UK-REACH study involved 17, 891 participants who consented to follow-up questionnaires and were recontacted via emailing professional bodies and social media. Initially, 6535 participants completed the third questionnaire, and 4246 participants completed the fourth questionnaire. Among these, 3248 participants completed both questionnaires. In total, 4137 HCWs reported having had COVID-19, but 104 were excluded for not providing ethnicity information. This resulted in a final cohort of 4033 HCWs who reported having had COVID-19, forming the analysis sample (Figure 1).

### Outcome measures

Our primary outcome was the presence or absence of long COVID symptom groups. Symptom duration was self-reported by participants in Questionnaire 3 and, where missing, supplemented with data from Questionnaire 4. Participants were asked to report the duration of each symptom using standardised response options (1–2 weeks, 2–4 weeks, 4–12 weeks or ⩾12 weeks). A binary variable was created to indicate whether any symptoms lasted ⩾12 weeks. Participants were classified as having long COVID if they reported at least one symptom persisting for ⩾12 weeks in either questionnaire, following a single or most recent episode of COVID-19 (details in Supplemental Material 2).

Both questionnaires included 32 symptoms of COVID-19. Three of these were free-text responses for ‘other’ symptoms to be reported by participants and were excluded from this analysis. Among the remaining 29 symptoms, one symptom (skin rash) had a negligible prevalence (*n* = 3) and was thus excluded from the analysis due to insufficient cases for meaningful statistical interpretation. We grouped the remaining 28 symptoms into seven groups based on existing previous literature:^14–16^ (cardiopulmonary, gastrointestinal, musculoskeletal, neurocognitive and neurologic, upper respiratory tract, psychological and social, and systemic) as shown in Table 1.

**Table 1.** Grouping of long COVID symptoms.

Cardiopulmonary	Gastrointestinal	Musculoskeletal	Neurocognitive and neurologic	Upper respiratory tract	Psychological and social	Systemic
Cough – dry	Nausea/vomiting	Muscle or body aches	Difficulty concentrating	Runny nose or nasal congestion	Feeling anxious	Chills or shivering
Cough – productive of mucus or phlegm	Diarrhoea	Joint pain	Memory loss or confusion	Sore throat	Feeling depressed	Fever
Shortness of breath	Abdominal pain		Tinnitus (ringing or noises in the ears)	Sneezing	Difficulty sleeping	Reduced appetite
Chest pain or tightness			Pins and needles or numbness			Fatigue
Palpitations			Headache			Dizziness
Loss or change in sense of smell						
Loss or change in sense of taste						

Our secondary outcome was long COVID (to address our objective of examining symptom patterns between HCWs with and without long COVID), a binary measure of the presence or absence of long COVID among HCWs reporting having had COVID-19.

Long COVID was defined as symptoms that persisted for ⩾12 weeks following a SARS-CoV-2 infection to align with the NICE definition of long COVID.^4^ A long COVID variable was derived using data from the third and fourth questionnaires based on symptom duration, where participants reporting symptoms lasting ⩾12 weeks were classified as having long COVID. If a participant had no symptoms or symptoms lasting less than 12 weeks in both questionnaires, they were classified as not having had long COVID, with preference given to the third questionnaire data, unless unavailable or more recent data were present in the fourth questionnaire.

See Supplemental Material 2 for the derivation of the outcome variable.

Our cohort consisted of participants who reported being infected with COVID-19. A participant’s history of COVID-19 was derived from answers to the question ‘Do you think that you currently have or have had COVID-19?’. Those who answered ‘Yes, my own suspicions’, ‘Yes, suspected by a doctor but not tested’ or ‘Yes, confirmed by a positive test’ were considered to have had COVID-19.

### Exposure

We had multiple exposures of interest. These included self-reported ethnicity, which we classified according to the 5- and 18-level ethnic group categories provided by the UK’s Office for National Statistics (ONS).^17^ We used the five-level variable (White, Asian, Black, Mixed and Other) in the primary analysis to balance maximising statistical power for detecting differences between ethnic groups with heterogeneity within each ethnic group. In addition, job roles were categorised into five groups: (1) doctor or medical support, (2) nurse, nursing assistant or midwife, (3) allied health professional (AHP), including pharmacists, healthcare scientists, ambulance workers and those in optical roles, (4) dental professionals and (5) administrative, estates or other roles. Clinical factors were also considered, including the number of COVID-19 vaccine doses at the time of acute infection (categorised as 0, 1, 2 or 3 doses).

### Covariates

We adjusted our multivariable models for a set of key confounders, including age and sex, which were hypothesised to confound each of the exposure/outcome relationships.

Supplemental Material 3 contains a description of each variable, which questionnaire(s) it was measured in, and how it was derived (if applicable).

### Statistical analysis

We excluded those with missing data for the primary exposure and outcome of interest from all analyses. We were primarily interested in the prevalence of the most common long COVID symptom groups and in determining factors associated with long COVID symptom groups in those with a history of acute COVID-19 infection and in the association of ethnicity with these symptoms. Analyses of factors associated with individual long COVID symptom groups were restricted to participants with long COVID. For each logistic regression model, the outcome was the presence or absence of a specific symptom group.

We summarised categorical variables as frequency and percentage, and non-normally distributed continuous variables as median (interquartile range [IQR]). We compared long COVID symptom groups between ethnic groups using chi-square tests, as they were categorical variables. We used univariable and multivariable logistic regression to determine unadjusted and adjusted associations of the variables described above with long COVID and its groups and reported results as unadjusted and adjusted odds ratios (ORs and aORs) and 95% confidence intervals (95% CIs).

We reported the frequency and percentage of observations with missing data for each variable of interest. We stratified the analysed cohort by ethnicity, together with tests of association between symptom profiles and ethnicity.

We used multiple imputation by chained equations to impute missing data in these logistic regression models.^18^ We also used Rubin’s rules to combine the parameter estimates and standard errors from 10 imputations into a single set of results.^19^

The imputation models used in the final analyses included all variables, including the outcome measure.

To investigate the extent to which differences in long COVID risk by ethnic group and occupational role could be explained by other related risk factors, we generated a base logistic regression model (i.e. the minimally adjusted model), in which we adjusted for minimum basic demographic variables, which are confounders (i.e. age, sex and migration status). This step ensures that any observed differences are more likely attributable to ethnicity rather than underlying demographic influences.^20,21^ Then, we included mediators as additional variables into this base model, which we called the ‘Fully-adjusted model’ that included age, sex, migration status and vaccination status. Comparing the minimally adjusted and fully adjusted models before and after including vaccination status helped us assess the extent to which vaccination disparities contribute to the observed differences in long COVID risk across ethnic groups.

To assess the potential impact of imputing missing data on our results, we conducted a sensitivity analysis using only observations with complete data in all covariates. This analysis aimed to evaluate the robustness of our findings when dealing with missing data.

We conducted all analyses and multiple imputation using Stata 18 (StataCorp. 2023. Stata Statistical Software: Release 18. College Station, TX: StataCorp LLC.).^22^

### Patient and public involvement

We worked closely with a Professional Expert Panel of HCWs from diverse ethnic and occupational backgrounds with long COVID who helped shape the research question and design the questionnaires of this study.

## Results

### Description of the analysed cohort

A description of the analysed cohort (*N* = 4033) is shown in Table 2.

**Table 2.** Description of the analysed cohort.

Variable	Analysed cohort Those who were infected with COVID-19 *N* = 4033	No long COVID (short COVID-19) (2966/4033) (73.5%)	Long COVID (1067/4033) (26.5%)
Ethnicity			
White	3038 (75.3%)	2167 (73.1%)	871 (81.6%)
Asian	648 (16.1%)	523 (17.6%)	125 (11.7%)
Black	133 (3.3%)	108 (3.6%)	25 (2.3%)
Mixed	153 (3.8%)	117 (3.94%)	36 (3.4%)
Other	61 (1.5%)	51 (1.72%)	10 (0.9%)
Missing	0 (0.0%)	0 (0.0%)	0 (0.0%)
Migration status			
Born in the UK	3160 (78.4%)	2298 (77.5%)	862 (80.8%)
Born abroad	868 (21.5%)	665 (22.4%)	203 (19.0%)
Missing	5 (0.1%)	3 (0.1%)	2 (0.2%)
Age, median (IQR)	45 (35–55)	44 (35–54)	49 (38–55)
Missing	396 (9.8%)	296 (10.0%)	100 (9.4%)
Sex			
Male	847 (21.0%)	674 (22.7%)	173 (16.2%)
Female	2798 (69.4%)	2001 (67.5%)	979 (74.7%)
Missing	388 (9.6%)	291 (9.8%)	97 (9.1%)
No. of COVID vaccines at the time of acute infection			
0	1512 (37.5%)	909 (30.7%)	603 (56.5%)
1	162 (4.0%)	115 (3.9%)	47 (4.4%)
2	353 (8.8%)	278 (9.4%)	75 (7.0%)
3	1511 (37.5%)	1335 (45.0%)	176 (16.5%)
Missing	495 (12.3%)	329 (11.1%)	166 (15.6%)
Type of COVID variant			
Wuhan	1600 (39.7%)	952 (32.1%)	648 (60.7%)
Alpha	242 (6.0%)	160 (5.4%)	82 (7.7%)
Delta	559 (13.9%)	438 (14.8%)	121 (11.3%)
Omicron BA.1	417 (10.3%)	354 (11.9%)	63 (5.9%)
Later Omicron subvariant	966 (24.0%)	882 (29.7%)	84 (7.9%)
Missing	249 (6.2%)	180 (6.1%)	69 (6.5%)
Severity of acute COVID-19 infection			
Not bed or sofa-bound	960 (23.8%)	846 (28.5%)	114 (10.7%)
In bed or on the sofa for 1–3 days	1200 (29.8%)	980 (33.0%)	220 (20.6%)
In bed or on the sofa for 4–6 days	677 (16.8%)	459 (15.5%)	218 (20.4%)
In bed or on the sofa for 7 days or longer	703 (17.4%)		703 (17.4%)
Missing	493 (12.2%)	416 (14.0%)	77 (7.2%)
Index of multiple deprivation quintile			
1 (most deprived)	312 (7.7%)	225 (7.6%)	87 (8.2%)
2	522 (12.9%)	393 (13.3%)	129 (12.1%)
3	671 (16.6%)	482 (16.3%)	189 (17.7%)
4	785 (19.5%)	586 (19.8%)	199 (18.7%)
5 (least deprived)	946 (23.5%)	699 (23.6%)	247 (23.2%)
Missing	797 (19.8%)	581 (19.6%)	216 (20.2%)
Co-morbidities			
Not diabetic	3197 (79.3%)	2363 (79.7%)	834 (78.2%)
Diabetic	112 (2.8%)	78 (2.6%)	34 (3.2%)
Missing	724 (18.0%)	525 (17.7%)	199 (18.7%)
Co-morbidities			
No depression	2906 (72.1%)	2198 (74.1%)	708 (66.4%)
Depression	416 (10.3%)	250 (8.4%)	166 (15.6%)
Missing	711 (17.6%)	518 (17.5%)	193 (18.1%)
Co-morbidities			
No anxiety	2740 (67.9%)	2095 (70.6%)	645 (60.5%)
Anxiety	592 (14.7%)	361 (12.2%)	231 (21.7%)
Missing	701 (17.4%)	510 (17.2%)	191 (17.9%)
Co-morbidities			
Not asthmatic	2886 (71.6%)	2184 (73.6%)	702 (65.8%)
Asthmatic	425 (10.5%)	259 (8.7%)	166 (15.6%)
Missing	722 (17.9%)	523 (17.6%)	199 (18.7%)
Co-morbidities			
No other lung conditions	3279 (81.3%)	2432 (82.0%)	847 (79.4%)
Other lung conditions	31 (0.8%)	9 (0.3%)	22 (2.1%)
Missing	723 (17.9%)	525 (17.7%)	198 (18.7%)
Co-morbidities			
No other CVDs^a^	3638 (90.2%)	2705 (91.2%)	933 (87.4%)
Other CVDs	365 (9.1%)	240 (8.1%)	125 (11.7%)
Missing	30 (0.74%)	21 (0.7%)	9 (0.8%)
Body mass index			
<25 ⩾25 and <30	1577 (39.1%) 916 (22.7%)	1226 (41.3%) 666 (22.5%)	351 (32.9%) 250 (23.4%)
⩾30 and <40	536 (13.3%)	362 (12.2%)	174 (16.3%)
⩾40	80 (2.0%)	43 (1.5%)	37 (3.5%)
Missing	924 (22.9%)	669 (22.6%)	255 (23.9%)
Occupation			
Doctor or medical support	811 (20.1%)	668 (22.5%)	143 (13.4%)
Nurse, NA or midwife	817 (20.3%)	535 (18.0%)	282 (26.4%)
Allied health professional*	1550 (38.4%)	1132 (38.2%)	418 (39.2%)
Dental	207 (5.1%)	157 (5.3%)	50 (4.7%)
Admin, estates or other	203 (5.0%)	137 (4.8%)	66 (6.2%)
Missing	445 (11.0%)	331 (11.4%)	108 (10.1%)

95% Cl: 95% confidence interval; OR: odds ratio.

a Heart diseases, heart problems, stroke, hypertension (HTN).

* Also includes pharmacists, healthcare scientists, ambulance workers and those in optical roles.

The majority of participants (69.4%) were females, and the median age was 45 years (IQR: 35–55); 24.7% of the cohort were from an ethnic minority group (16.1% Asian, 3.8% Mixed, 3.3% Black and 1.5% Other); 21.5% were born outside the UK. The largest occupational group was AHPs (38.4%), followed by nursing roles (20.3%) and doctors or medical support staff (20.1%), with 5.1% in dental roles, and 5.0% in administrative or other roles. At the time of the reported acute infection, 37.5% had received no COVID-19 vaccines, 4% had one dose, 8.8% two doses and 37.5% had three doses (Table 2). A higher proportion of participants with long COVID had not received any COVID-19 vaccination at the time of their acute infection (56.5%) compared with those without long COVID (30.7%), as shown in Table 2.

### Symptom patterns and differences among HCWs with long COVID and without long COVID

**Figure 2.**
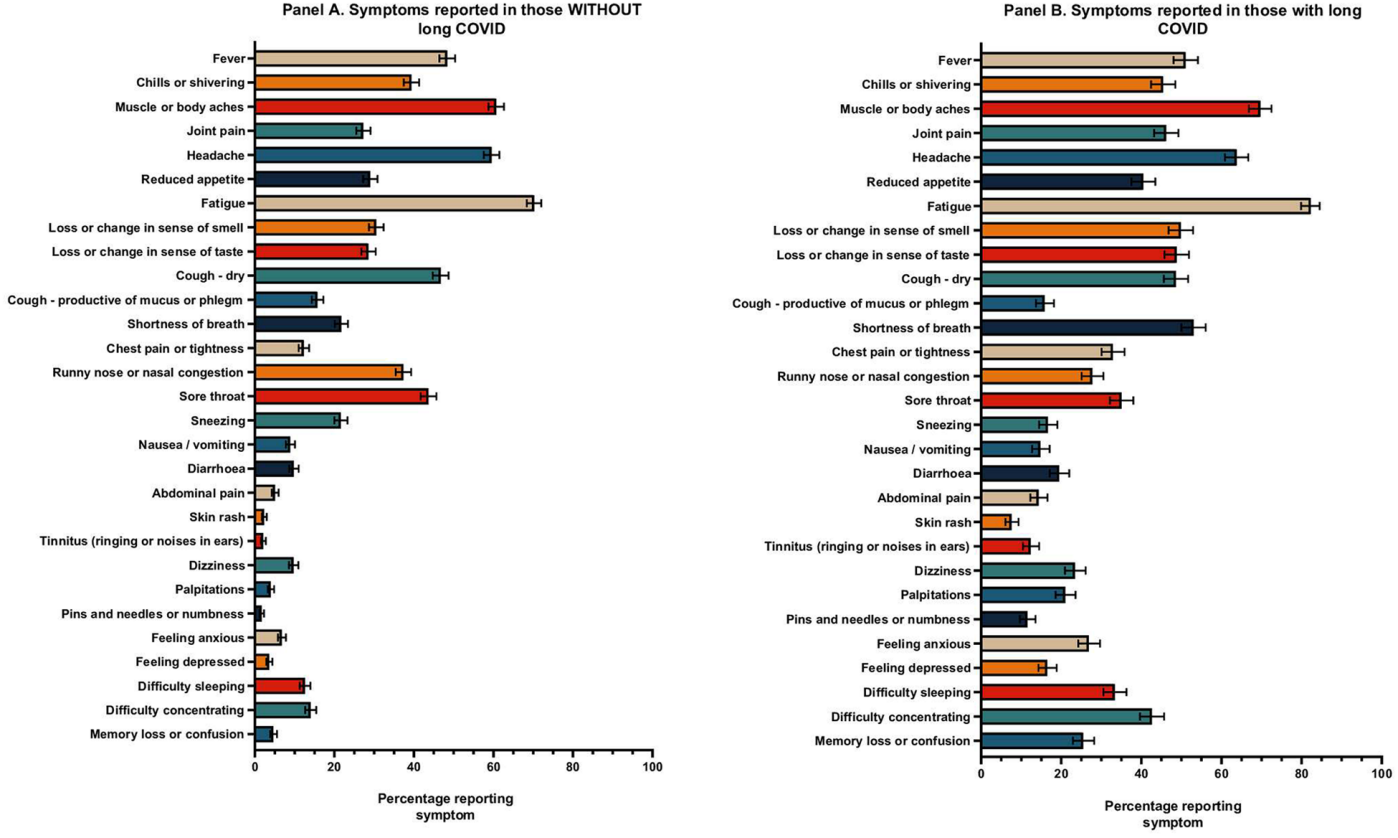
Symptom patterns differ among HCWs with long COVID and without groups of long COVID symptoms and differences by ethnicity.

Figure 2 presents descriptive symptom patterns and differences among HCWs with and without long COVID. No statistical comparisons were performed for these differences, as this was not a primary objective of the study.

Overall, there is a greater prevalence of symptoms reported by HCWs with long COVID compared with those without long COVID. This is particularly apparent in systemic symptoms such as fatigue, neurologic symptoms including pins and needles or numbness, difficulty sleeping, difficulty concentrating and memory loss or confusion. Additionally, there is a higher reporting of psychological symptoms, such as depression and anxiety, among those with long COVID compared with those without long COVID.

Supplemental material 4 presents the distribution of symptom groups by ethnicity.

Among the 1067 number of HCWs that reported long COVID, the most common symptom groups were neurocognitive and neurologic (63.4%), cardiopulmonary (40.0%, with the highest prevalence among Asian HCWs at 45.6%), systemic (54.6%, particularly affecting Black and Mixed ethnicities at 64.0% and 63.9%, respectively) and musculoskeletal (32.2%, with the highest prevalence among Mixed ethnicity at 30.6%).

**Figure 3.**
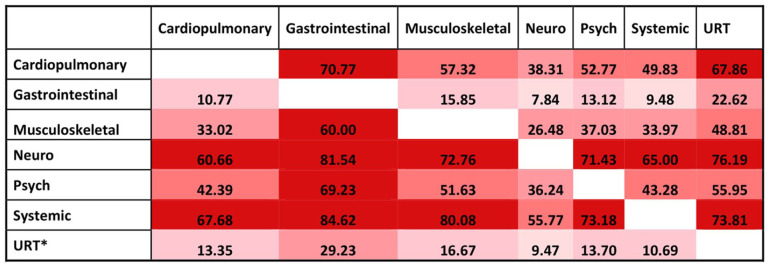
Overlap between long COVID symptom groups among HCWs with long COVID. Values are the percentage of participants with the column symptom group who also reported the row group. *URT: upper respiratory tract.

There was a co-occurrence across symptom groups (Figure 3). The highest overlaps were observed between neurocognitive/neurologic and psychological/social symptoms, and between systemic and cardiopulmonary symptoms. Overlap was also common between musculoskeletal and systemic symptoms. These patterns indicate that many participants reported symptoms across multiple groups.

### Association of ethnicity and other factors with the risk of experiencing long COVID symptom groups

#### Univariable analysis

Supplemental Material 5 presents the unadjusted ORs for the association of ethnicity with experiencing long COVID symptoms. There were no significant differences in the risk of experiencing particular symptom groups by ethnic group.

#### Multivariable analysis

**Figure 4.**
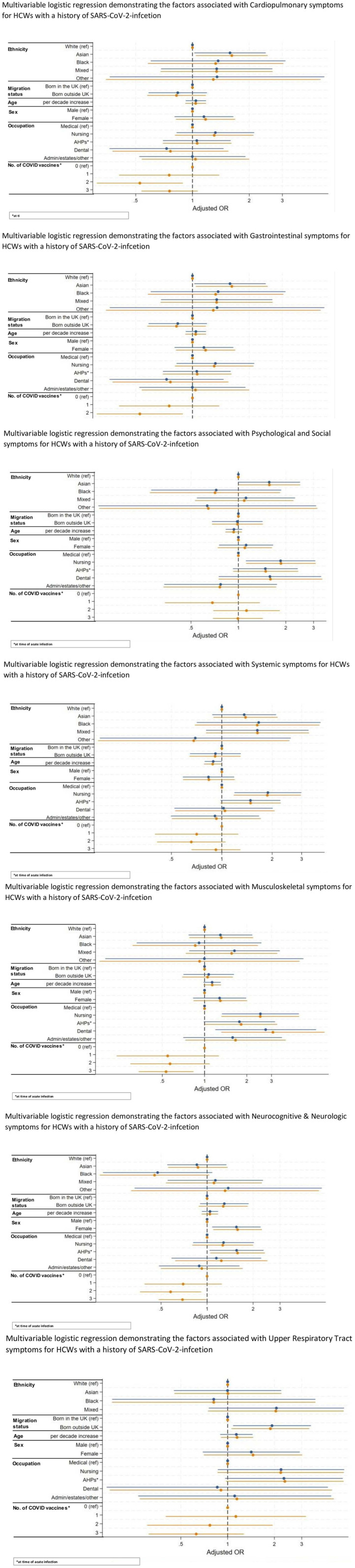
Multivariable logistic regression demonstrating the factors associated with each long COVID symptom group for HCWs with a history of SARS-CoV-2 infection.

Figures 4 show multiple logistic regression demonstrating the factors associated with each of the long COVID groups for HCWs with a history of COVID-19.

### Multivariable logistic regression demonstrating

#### Minimally adjusted model

On multivariable analysis in a minimally adjusted model where we adjusted for basic demographics such as age, sex, migration status and occupation, it was found that HCWs from Asian ethnicity were at significantly higher odds of experiencing cardiopulmonary symptoms (aOR: 1.58, 95% CI 1.02–2.45, *p* = 0.039), compared with their White counterparts. Female HCWs were more likely to experience gastrointestinal symptoms (aOR: 3.74, 95% CI 1.14–12.32, *p* = 0.030). HCWs who are in nursing and dental roles were more likely to experience musculoskeletal symptoms, while female and AHPs were more likely to experience neurocognitive and neurologic symptoms. HCWs in nursing roles were also more likely to experience psychological and social as well as systemic symptoms, and those born abroad were more likely to experience upper respiratory tract symptoms (aOR: 1.93, 95% CI 1.08–3.44, *p* = 0.026), as shown in Figure 4.

#### Fully adjusted model

**Figure 5.**
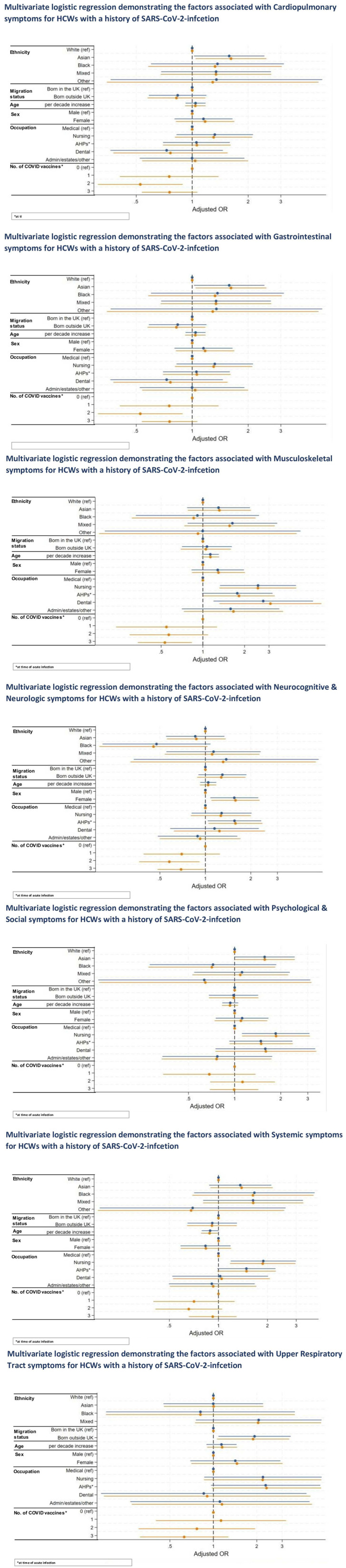
Multivariable logistic regression demonstrating the factors associated with each long COVID symptom group for HCWs with a history of SARS-CoV-2 infection.

On multivariable analysis in a fully adjusted model (see Figure 5), we found that female HCWs were almost four times more likely to experience gastrointestinal symptoms (aOR: 3.78, 95% CI 1.14–12.45, *p* = 0.029) than males. They were also more likely to report neurocognitive and neurologic symptoms (aOR: 1.58, 95% CI 1.10–2.28, *p* = 0.014). As for occupational roles, for musculoskeletal symptoms, HCWs in nursing (aOR: 2.50, 95% CI 1.32–4.72, *p* = 0.005), allied health professional (aOR: 1.82, 95% CI 1.01–3.30, *p* = 0.048) and dental roles (aOR: 3.07, 95% CI 1.31–7.17, *p* = 0.010) were more likely to be affected than those in medical roles. Additionally, AHPs were more likely to experience neurocognitive and neurologic symptoms (aOR: 1.58, 95% CI 1.05–2.39, *p* = 0.029) compared with those in medical roles. Nursing roles were also more likely to experience psychological and social symptoms, and systemic symptoms.

Asian ethnicity was significantly associated with an increased likelihood of cardiopulmonary symptoms (aOR: 1.62, 95% CI 1.04–2.51, *p* = 0.032). Furthermore, HCWs who were born abroad had higher odds of experiencing upper respiratory tract symptoms (see Figure 4). Same as in the minimally adjusted model, older age was a significant risk factor for systemic symptoms.

In terms of vaccination status, two or three COVID-19 vaccine doses were associated with a lower likelihood of experiencing cardiopulmonary symptoms. Two or three doses were similarly linked to a reduced risk of neurocognitive and neurologic symptoms, while specifically having three doses was associated with decreased odds of musculoskeletal symptoms compared with having no COVID-19 vaccination.

### Sensitivity analyses

Analysing observations with complete data in all covariates for the univariable analysis, fully adjusted and minimally adjusted models (see Supplemental Material 6) did not significantly alter the interpretation of the results of the primary analysis (i.e., the significant predictors remained the same).

## Discussion

### Summary of principal findings in relation to other studies

We present the first findings from the largest nationwide study in the UK to investigate the most common symptoms of long COVID, their patterns, and how they vary by sociodemographic, clinical and occupational factors among HCWs across the UK. We found that for patterns of symptoms of long COVID among HCWs compared with those without long COVID, HCWs with long COVID had a higher prevalence of systemic, neurological and psychological symptoms compared with those without long COVID. This aligns with findings from a narrative review, which highlighted that HCWs are at increased risk of long COVID, with common symptoms including fatigue, cognitive dysfunction and psychological distress.^23^

In the general population, studies have reported similar symptom patterns among individuals with long COVID. The UK’s Office for National Statistics (ONS) estimated that approximately two million people were experiencing self-reported long COVID as of January 2023, with fatigue, difficulty concentrating and depression among the most common symptoms.^24^

In our study, Asian HCWs were more likely to report cardiopulmonary symptoms, such as breathlessness and chest pain, compared with HCWs from White ethnicity. Conversely, White HCWs more frequently reported fatigue and cognitive symptoms, such as ‘brain fog’, which could suggest that symptom expression and reporting are influenced by cultural factors. For example, cardiopulmonary symptoms might be more readily associated with severe illness in some cultures, prompting higher reporting rates among Asian HCWs. Conversely, cognitive symptoms may be underreported due to stigma or lack of recognition.^25^ Further research is needed to understand whether these differences reflect true variations in symptom prevalence or disparities in healthcare-seeking behaviour and reporting practices. A 2022 report by the ONS revealed that the Asian ethnic group is the second largest ethnic group in England and Wales, making up 9.3% of the population (59.6 million).^26^ In England, South Asians have a higher prevalence of existing co-morbidities such as diabetes, compared with White individuals.^27^ In addition, Asian populations present with the highest proportion of sudden arrhythmic death to Sudden Cardiac Death when compared with Black, Hispanic and White populations.^28^ This may partially explain the cardiopulmonary health disparities in long COVID outcomes, particularly among Asian individuals.^29^ These pre-existing health differences may also predispose certain ethnic groups to experience symptoms independent of COVID-19 itself, which could confound the attribution of symptoms to long COVID. Future studies should consider including pre-COVID health status or baseline symptom reporting to help disentangle these effects.

The literature on symptom variation by ethnicity is still evolving and remains unclear. For example, one study found that individuals from Black ethnic backgrounds were more likely to report memory problems, while Hispanics were more likely to report difficulties in understanding, compared with White individuals.^30^ Another US study, based on 711 telephone interviews conducted at least 2 months post-COVID-19-positive test, found that Black or African American (Black) individuals were at higher risk of reporting any long COVID symptom, particularly dyspnoea and arthralgia/myalgia, compared with other ethnic groups, including White individuals.^31^ Similarly, several studies from the US, UK and the Netherlands reported that ethnic minority groups, including African, Asian and Turkish individuals, were at higher risk of reporting long COVID symptoms than native majority populations in these countries.^32–35^

These differences may be partly attributed to routine electronic database studies failing to capture cultural and sociodemographic aspects of symptom presentations and variations in recruited populations. Research has highlighted inter-ethnic differences in symptoms like musculoskeletal pain.^36^ Significant variations have also been observed within groups categorised as ethnic minorities, such as South Asians, due to the heterogeneity within these populations.^37^

In terms of other factors, HCWs in nursing roles were associated with a higher likelihood of psychological, social and systemic symptoms. This could reflect the unique stressors and workload pressures faced by nurses, which are well documented in the literature.^38,39^ Nurses often experience higher exposure to infectious diseases, compounded by work stress, which may exacerbate the severity and range of long COVID symptoms.^40,41^ Similarly, the increased likelihood of musculoskeletal symptoms among HCWs in nursing and dental roles is consistent with findings from prior research linking these professions to physical strain, particularly during the pandemic.^42^

The gender disparities observed in the study, with female HCWs being disproportionately affected by neurocognitive, neurologic and gastrointestinal symptoms, which shows that females are more likely to experience persistent symptoms post-COVID-19, are consistent across most studies looking at associations with long COVID, including both the general population and HCWs.^43–48^ This could be attributable to sex-based differences in immune responses and hormonal influences, which may affect symptom manifestation, differences in reporting and recovery.^49,50^

The association between older age and systemic symptoms aligns with previous studies demonstrating that an age-related decline in immune function and increased co-morbidities contribute to worse outcomes and prolonged symptomatology in older adults.^51^

Finally, the protective role of vaccination, as evidenced in our findings by reduced likelihood of cardiopulmonary, musculoskeletal and neurocognitive symptoms, aligns with findings from Antonelli et al. on the general population,^52^ and from Foulkes et al., who found that vaccinated HCWs reported fewer persistent symptoms.^53^ This underscores the importance of vaccine uptake among HCWs as a strategy to reduce the burden of long COVID and support workforce sustainability, particularly among ethnic minority groups.

### Strengths and weaknesses of the study

This study employed a robust methodological approach by using multiple questionnaires to capture accurate information on vaccination status, long COVID symptoms and other variables. This ensured high data completeness and reliability. Another strength is that we included a diverse sample of HCWs from various ethnic backgrounds with specific data on their migration status, which allowed for an in-depth analysis of how symptom profiles vary across different ethnic groups. However, this study has limitations. For instance, this is self-reported data, which is subject to recall bias and might influence respondents to choose a more socially acceptable answer rather than being truthful. Moreover, a cross-sectional study design limits the investigation of the direction of causality. Nonetheless, using both minimally and fully adjusted models provides valuable insights into the independent effects of various demographic and occupational factors on symptom presentation.

As this was a secondary analysis of a pre-existing cohort study, formal a priori power calculations for subgroup analyses were not possible. However, we included a large sample of HCWs with a history of COVID-19 (*n* = 4033), of whom over 1000 had long COVID, which provides a strong foundation for identifying meaningful trends. Nevertheless, while some associations reached statistical significance, their practical or clinical relevance may be limited. As such, the findings should be considered exploratory rather than definitive, and should be interpreted in the context of the wider evidence base. In addition, conducting separate analyses for each of the seven symptom groups introduces the issue of multiple testing. Although our findings are hypothesis-generating, they should be interpreted with caution and confirmed in future studies with appropriate correction methods.

Additionally, the combination of data from two questionnaires administered several months apart, while necessary to increase sample size and data completeness, introduces the possibility of information bias. During the inter-survey period, there was substantial public and professional discourse about long COVID. Given the high health literacy and media exposure of HCWs, this evolving narrative may have influenced how participants perceived and reported their symptoms, particularly in the later survey. This raises the possibility of reporting bias where responses may be shaped by heightened awareness or social framing rather than solely by biological experience. Furthermore, the extended period over which data were collected introduces potential variability in symptom reporting, vaccination status and other factors (e.g. COVID-19 variants in circulation, public health policies or healthcare workloads). These time-related differences may affect comparability between participants and complicate the interpretation of symptom duration and prevalence. Therefore, results should be interpreted with caution. Furthermore, our models investigating associations with individual symptom groups were restricted to participants with long COVID, comparing those who did versus did not report a particular symptom group. However, individuals may have reported symptoms across multiple groups, meaning that the comparator group may include those with other long COVID symptom profiles.

Another limitation lies in including both laboratory-confirmed and self-reported COVID-19 cases. While this approach aimed to capture a broad spectrum of HCWs potentially affected by long COVID, particularly given limitations in test access during earlier stages of the pandemic, it also risks misclassification bias. However, this pragmatic inclusion reflects the real-world limitations of testing during key phases of the pandemic, and aligns with other large-scale epidemiological studies using similar criteria.^54^

## Conclusion and future research

In conclusion, this study provided valuable insights into symptom grouping and patterns among HCWs, revealing that those with long COVID experienced a higher prevalence of systemic, neurological and psychological symptoms compared with those without long COVID. Distinct long COVID symptom groups, such as neurocognitive, cardiopulmonary and musculoskeletal symptoms, were observed, with some variation in prevalence across ethnic groups and occupational roles. These findings underscore the need for enhanced data collection on long COVID symptoms prevalence and differences by occupational, ethnic and clinical factors. Moreover, the significant associations we found between demographic factors and specific symptom groups highlight the importance of targeted support strategies. Future research should focus on longitudinal studies to establish causal relationships and temporal dynamics of association. Additionally, our findings emphasise the need for workplace adjustments that consider both occupation-specific risks and individual sociocultural factors, ensuring that return to work strategies are both safe and productive.

## Supplemental Material

sj-docx-1-jrs-10.1177_01410768251389692 – Supplemental material for Patterns of long COVID symptoms among healthcare workers in the UK and variations by sociodemographic, clinical and occupational factors: a cross-sectional analysis of a nationwide study (UK-REACH) Supplemental material, sj-docx-1-jrs-10.1177_01410768251389692 for Patterns of long COVID symptoms among healthcare workers in the UK and variations by sociodemographic, clinical and occupational factors: a cross-sectional analysis of a nationwide study (UK-REACH) by Amani Al-Oraibi, Christopher A Martin, Katherine Woolf, Laura B Nellums, Carolyn Tarrant and Manish Pareek in Journal of the Royal Society of Medicine

## Declarations

**Authors’ note:** On behalf of the UK-REACH Study Collaborative Group+ +Manish Pareek (Chief investigator, University of Leicester), Laura Gray (University of Leicester), Laura Nellums (University of New Mexico), Anna L Guyatt (University of Leicester), Catherine John (University of Leicester), I Chris McManus (University College London), Katherine Woolf (University College London), Ibrahim Abubakar (University College London), Amit Gupta (Oxford University Hospitals), Avinash Aujayeb (Northumbria Specialist Emergency Care Hospital), Bindu Gregary (Royal Preston Hospital), Rubina Reza (Derbyshire Healthcare NHS Foundation Trust), Sandra Simpson (Nottinghamshire Healthcare NHS Foundation Trust), Stephen Zingwe (Berkshire Healthcare NHS Foundation Trust), Keith R Abrams (University of York), Martin D Tobin (University of Leicester), Louise Wain (University of Leicester), Sue Carr (University Hospitals of Leicester NHS Trust), Edward Dove (University of Edinburgh), Kamlesh Khunti (University of Leicester), David Ford (University of Swansea) and Robert Free (University of Leicester).

**Ethics approval:** This study received appropriate ethical approval from the London – Brighton and Sussex Research Ethics Committee (REC) and Health Research Authority REC (Ref No 20/HRA/4718). All participants provided informed consent prior to their participation, and their confidentiality and anonymity were maintained throughout the study. Participants were informed of their right to withdraw at any time without consequence.

**Guarantor:** MP.
