# Supplementary material for: Patterns of long COVID symptoms among healthcare workers in the UK and variations by sociodemographic, clinical and occupational factors: a cross-sectional analysis of a nationwide study (UK-REACH)
Source: J R Soc Med. 2025 Dec 8;118(12):387–406. doi: 10.1177/01410768251389692 (PMC12685703; doi:10.1177/01410768251389692)
Supplement: sj-docx-1-jrs-10.1177_01410768251389692 – Supplemental material for Patterns of long COVID symptoms among healthcare workers in the UK and variations by sociodemographic, clinical and occupational factors: a cross-sectional analysis of a nationwide study (UK-REACH) [file sj-docx-1-jrs-10.1177_01410768251389692.docx]

**SUPPLEMENTARY MATERIAL 1. STROBE Statement—checklist of items that should be included in reports of observational studies**

|  | **Item No** | **Recommendation** | **Page no.** |
| --- | --- | --- | --- |
| **Title and abstract** | 1 | (*a*) Indicate the study’s design with a commonly used term in the title or the abstract | 1,2 |
|  |  | (*b*) Provide in the abstract an informative and balanced summary of what was done and what was found | 2 |
| **Introduction** | | |  |
| Background/rationale | 2 | Explain the scientific background and rationale for the investigation being reported | 3 |
| Objectives | 3 | State specific objectives, including any prespecified hypotheses | 4 |
| **Methods** | | |  |
| Study design | 4 | Present key elements of study design early in the paper | 5 |
| Setting | 5 | Describe the setting, locations, and relevant dates, including periods of recruitment, exposure, follow-up, and data collection | 5 |
| Participants | 6 | (*a*) Give the eligibility criteria, and the sources and methods of selection of participants | 5 |
| Variables | 7 | Clearly define all outcomes, exposures, predictors, potential confounders, and effect modifiers. Give diagnostic criteria, if applicable | 6,7 |
| Data sources/  measurement | 8* | For each variable of interest, give sources of data and details of methods of assessment (measurement). Describe comparability of  assessment methods if there is more than one group | SM2 |
| Bias | 9 | Describe any efforts to address potential sources of bias | 8 |
| Study size | 10 | Explain how the study size was arrived at | 9 |
| Quantitative variables | 11 | Explain how quantitative variables were handled in the analyses. If applicable, describe which groupings were chosen and why | 7,8 |
| Statistical methods | 12 | (*a*) Describe all statistical methods, including those used to control for confounding | 8 |
|  |  | (*b*) Describe any methods used to examine subgroups and interactions | 8 |
|  |  | (*c*) Explain how missing data were addressed | 8 |
|  |  | (*d*) If applicable, describe analytical methods taking account of sampling strategy | NA |
|  |  | (*e*) Describe any sensitivity analyses | 8 |
| **Results** | | |  |
| Participants | 13* | (a) Report numbers of individuals at each stage of study—eg numbers potentially eligible, examined for eligibility, confirmed eligible, included in the study, completing follow-up, and analysed | 9,21 |
|  |  | (b) Give reasons for non-participation at each stage | 9 |
|  |  | (c) Consider use of a flow diagram | 21 |
| Descriptive data | 14* | (a) Give characteristics of study participants (eg demographic, clinical, social) and information on exposures and potential confounders | 9 |
|  |  | (b) Indicate number of participants with missing data for each variable of interest | 15 |
| Outcome data | 15* | Report numbers of outcome events or summary measures | 15 |
| Main results | 16 | (*a*) Give unadjusted estimates and, if applicable, confounder-adjusted estimates and their precision (eg, 95% confidence interval). Make clear which confounders were adjusted for and why they were included | 9,10 |
|  |  | (*b*) Report category boundaries when continuous variables were categorized | 9,10 |
|  |  | (*c*) If relevant, consider translating estimates of relative risk into absolute risk for a meaningful time period | NA |
| Other analyses | 17 | Report other analyses done—eg analyses of subgroups and interactions, and sensitivity analyses | 11 |
| **Discussion** | | |  |
| Key results | 18 | Summarise key results with reference to study objectives | 12 |
| Limitations | 19 | Discuss limitations of the study, taking into account sources of potential bias or imprecision. Discuss both direction and magnitude of  any potential bias | 14 |
| Interpretation | 20 | Give a cautious overall interpretation of results considering objectives, limitations, multiplicity of analyses, results from similar studies, and other relevant evidence | 14 |
| Generalisability | 21 | Discuss the generalisability (external validity) of the study results | 14 |
| **Other information** | | |  |
| Funding | 22 | Give the source of funding and the role of the funders for the present study and, if applicable, for the original study on which the  present article is based | 23 |

*Give information separately for cases and controls in case-control studies and, if applicable, for exposed and unexposed groups in cohort and cross-sectional studies.

**Note:** An Explanation and Elaboration article discusses each checklist item and gives methodological background and published examples of transparent reporting. The STROBE checklist is best used in conjunction with this article (freely available on the Web sites of PLoS Medicine at [http://www.plosmedicine.org/,](http://www.plosmedicine.org/) Annals of Internal Medicine at [http://www.annals.org/,](http://www.annals.org/) and Epidemiology at [http://www.epidem.com/).](http://www.epidem.com/)) Information on the STROBE Initiative is available at [www.strobe-statement.org.](http://www.strobe-statement.org/)

**SUPPLEMENTARY MATERIAL 2. Derivation of outcome variable**

- Symptom’s duration in questionnaire 3:
  - Participants were asked about the duration of each of the symptoms they experienced. The responses were categorised as follows:
    - 1 day to 2 weeks (1)
    - 2-4 weeks (2)
    - 4-12 weeks (3)
    - 12+ weeks (99)
  - Additionally, a response option indicated no symptoms at all (q3_c19sx___0).
- Identifying long COVID in questionnaire 3:
  - A binary variable ‘q3symplong12was’ was created to indicate if any of the symptoms lasted for ≥ 12 weeks (coded as 3 or more). If ‘q3_c19sx___0’ was 1 (no symptoms at all), ‘q3symplong12was’ was set to 0.
  - Another variable q3hadcov was generated to indicate if the participant had COVID-19, based on the condition that they have or have had COVID-19 (as explained above).
- Symptom duration in questionnaire 4:
  - Similar to questionnaire 3, participants reported the duration of their symptoms, with the same categorisation.
  - The binary variable q4symplong12was was created following the same logic as for Q3, and q4hadcov was generated to indicate if the participant had COVID-19 in Q4.
- Combining data from both questionnaires:
  - A participant was considered to have long COVID based on questionnaire 3 if they reported only one instance of COVID-19 and any symptoms lasting ≥ 12 weeks. This approach ensures information about COVID-19 is closest to the event. If this information was missing in questionnaire 3, it was taken from questionnaire 4. If a participant reported more than one episode of COVID-19, the information was taken from questionnaire 4 to use the most recent data.
  - A participant was considered not to have long COVID if they reported no symptoms lasting ≥ 12 weeks in both questionnaires 3 and 4.

**SUPPLEMENTARY MATERIAL 3.** **Derivation of covariates from questionnaire data**

| **Variable** | **Categorical/continuous** | **Description** | **Where is it derived from?** | **How is it derived?** |
| --- | --- | --- | --- | --- |
| **Ethnicity** | Categorical (non-ordinal) | Participants were asked to select their ethnicity from a list of the 18 Office for National Statistics categories:  Asian/Asian British – Indian  Asian/Asian British – Pakistani  Asian/Asian British – Bangladeshi  Asian/Asian British – Chinese  Asian/Asian British - Any other Asian background  Black/African/Caribbean/Black British - African  Black/African/Caribbean/Black British – Caribbean  Black/African/Caribbean/Black British - Any other Black/African/Caribbean background Mixed/Multiple ethnic groups - White and Black Caribbean  Mixed/Multiple ethnic groups - White and Black African  Mixed/Multiple ethnic groups - White and Asian  Mixed/Multiple ethnic groups - Any other Mixed/multiple ethnic background  White - English/Welsh/Scottish/Northern Irish/British  White – Irish  White - Gypsy or Irish Traveller  White - Any other white background  Other ethnic group – Arab  Other ethnic group - Any other ethnic background  These were categorised into the 5 broader Office for National Statistics ethnicity categories (Asian, Black, Mixed, White, Other). | From Q1, Q2, Q3 and Q4. | Ethnicity was derived from the same questionnaire used to report long COVID symptoms (>12 weeks). If ethnicity data were missing from this questionnaire (i.e., Q3 or Q4), it was taken from Q2 if possible and if missing in Q2 then from Q1. |
| **Migration status** | Categorical (binary) | Participants were asked whether they were born in the UK or born overseas. | From Q1, Q2, Q3 and Q4. | Migration status was derived based on the most common answer provided among the four questionnaires. |
| **Age** | Continuous | Age in years. Derived from date of birth entered by participants at registration. | From Q1, Q2, Q3 and Q4. | Age was divided by 10 to give age in decades. |
| **Sex** | Categorical (binary) | Participants were asked their sex assigned at birth. | From Q1, Q2, Q3 and Q4. | - |
| **No. of COVID vaccines at time of acute infection** | Categorical (ordinal) | In Q1 & Q2:  Participants were asked to select one of the following options on whether they have taken part in a trial of a COVID-19 vaccines:   - Yes - No, but I would if asked - No, but I would not if asked - Prefer not to answer   Participants were asked to select one of the following options on how many doses they have had:   - 1 - 2 - Unsure - Prefer not to answer   In Q3 & Q4:  For the first two doses, participants were asked to select one of the following options on how many doses they have had and enter date and month for each dose by selecting on of the following options:   - I have had both doses - I have had one dose only - I have had no doses - Prefer not to answer   For the third (booster) dose, participants were asked to select one of the following options on how many doses they have had and enter date and month for each dose by selecting on of the following options:     - Yes, had third/booster dose - Yes, and intend to have it - Yes, but undecided whether to have it - Yes, but decided not to have it - No, but intend to have it when offered - No, and undecided whether to have it when offered - No, and decided not to have it when offered - Prefer not to answer | From Q1, Q2, Q3 and Q4. | • First dose data has three sources of information (Q1, Q2, and Q3). Second dose data has two sources of information (Q2 and Q3). Third dose data has two sources of information (Q3 and Q4). As such, we have at least two sources of data for each dose to confirm and validate from.  • Once they have the vaccine administered, they are vaccinated (e.g., if they had two doses and they got infected at the time of the second dose, they are still considered to have had two doses).  • First, we derived a vaccine date for the first dose in which we had three sources of information allowing us to cross-check against a)what was feasible, b)what has been entered repeatedly. For the second dose date, we only had two sources of information. Where these dates agreed, that was taken as the date of the second dose, where they did not agree, we took the date that would’ve made sense with relation to their first dose. After deriving these two master variables, we coded as missing anyone for whom the second dose date preceded the first (N=23).  •First dose date adjustments from Q1:  Dec 2021 → Dec 2020  Jan 2020 → Jan 2021  Jan 2022 → Jan 2021  Dec 2022 → Dec 2020  Summer 2021 with trial participation → Summer 2020  •First dose dates after the completion date of the 1st questionnaire were marked as missing.  •Master vaccine variable for first dose date was first taken from Q1 because its closes to the event (to reduce recall bias).  •If the participant selected “No” or the value was missing for the trial question for both Q1 and Q2 and first dose vaccine date was before Dec 2020, it was coded as missing.  • If third dose date was before first trial date for the booster dose (i.e., Jun 2021), it was coded as missing.  • If the vaccine date and COVID infection date were in the same month, it was assumed the vaccine was administered first, due to the UK government policy against vaccination within 28 days of infection.^431^  • Finally, a master vaccine variable was generated using vaccine information across each questionnaire to validate vaccine date where possible. |
| **Occupation** | Categorical (non-ordinal) | Participants were asked to select their main job/role. Categorised as below:   - **Doctor or medical support** - Doctor, Advanced Critical Care Practitioner, Anaesthesia associate, Surgical Care Practitioner, Other medical associate - **Nurse, NA or Midwife -**  Advanced Nurse Practitioner, Healthcare assistant, Maternity support worker, Midwife, Nurse, Nursing Associate, Other nursing and midwifery role, - **Allied Health Professional (including pharmacists, ambulance workers and those in optical roles)** - Arts therapist, Biomedical scientist, Chiropodist/Podiatrist, Clinical scientist, Dietician, Hearing aid dispenser, Occupational therapist, Operating department practitioner, Orthoptist, Physiotherapist, Practitioner psychologist, Prosthetist / Orthotist, Radiographer, Speech and language therapist, Other Allied Health Professional role, Emergency medical , Paramedic , Other ambulance role, OT Support , Phlebotomist, Physiotherapy Assistant, Radiography Other clinical support role , Pharmacist , Pharmacy technician, Other pharmacy role, Optical - Dispensing optician, Optometrist, Other Optical role - **Dental -**  Clinical dental technician, Dental Hygienist, Dental nurse, Dental technician, Dentist, Other dental role - **Admin, estates or other –** Administration, Catering services, Domestic services, Estates services, Porter, Other | From Q1, Q2, Q3 and Q4. | A master occupation variable was generated to take the occupation variable from the questionnaire that the participant reports having had the longest episode of COVID-19 in. |

**SUPPLEMENTARY MATERIAL 4. Description of the analysed cohort stratified by ethnicity**

| **Long COVID symptom groups** | **Long COVID cohort**  **N=1,067** | | | | | |
| --- | --- | --- | --- | --- | --- | --- |
|  | **White** | **Asian** | **Black** | **Mixed** | **Other** | **P-value** |
| **Long COVID cohort**  **N=1,067** | **871 (81.6%)** | **125 (11.7%)** | **25 (2.3%)** | **36 (3.4%)** | **10 (0.9%)** | - |
| **No Cardiopulmonary**  **Cardiopulmonary** | 532 (61.1%)  339 (38.9%) | 68 (54.4%)  57 (45.6%) | 14 (56.0%)  11 (44.0%) | 20 (55.7%)  16 (44.4%) | 6 (60.0%)  4 (40.0%) | 0.641 |
| **No Gastrointestinal**  **Gastrointestinal** | 815 (93.6%)  56 (6.4%) | 120 (96.0%)  5 (4.0%) | 25 (100.0%)  0 (0.0%) | 32 (88.9%)  4 (11.1%) | 10 (100.0%)  0 (0.0%) | 0.289 |
| **No Musculoskeletal**  **Musculoskeletal** | 671 (77.0%)  200 (23.0%) | 97 (77.6%)  28 (22.4%) | 20 (80.0%)  5 (20.0%) | 25 (69.4%)  11 (30.6%) | 8 (80.0%)  2 (20.0%) | 0.851 |
| **No Neurocognitive and Neurologic**  **Neurocognitive and Neurologic** | 313 (35.9%)  558 (64.1%) | 50 (40.0%)  75 (60.0%) | 13 (52.0%)  12 (48.0%) | 12 (33.3%)  24 (66.7%) | 3 (30.0%)  7 (70.0%) | 0.449 |
| **No Psychological and Social**  **Psychological and Social** | 596 (68.4%)  275 (31.6%) | 77 (61.6%)  48 (38.4%) | 19 (76.0%)  6 (24.0%) | 24 (66.7%)  12 (33.3%) | 8 (80.0%)  2 (20.0%) | 0.843 |
| **No systemic**  **Systemic** | 406 (46.6%)  465 (53.4%) | 53 (42.4%)  72 (57.6%) | 9 (36.0%)  16 (64.0%) | 13 (36.1%)  23 (63.9%) | 6 (60.0%)  4 (40.0%) | 0.413 |
| **No Upper Respiratory Tract**  **Upper Respiratory Tract** | 804 (92.3%)  67 (7.7%) | 115 (92.0%)  10 (8.0%) | 23 (92.0%)  2 (8.0%) | 31 (86.1%)  5 (13.9%) | 10 (100.0%)  0 (0.0%) | 0.610 |

SUPPLEMENTARY MATERIAL 5. The Unadjusted ORs for the association of ethnicity with experiencing long COVID symptoms.

| **Variable** | **Total N=1,067** | | | | | | | | | | | | | |
| --- | --- | --- | --- | --- | --- | --- | --- | --- | --- | --- | --- | --- | --- | --- |
|  |  |  |  |  |  |  |  |  |  |  |  |  |  |  |
|  |  |  |  |  |  |  |  |  |  |  |  |  |  |  |
|  | **Cardiopulmonary** | | **Gastrointestinal** | | **Musculoskeletal** | | **Neurocognitive & Neurologic** | | **Psychological & Social** | | **Upper Respiratory Tract** | | **Systemic** | |
|  | **Unadjusted**  **OR (95% Cl)** | **P-value** | **Unadjusted**  **OR (95% Cl)** | **P-value** | **Unadjusted**  **OR (95% Cl)** | **P-value** | **Unadjusted**  **OR (95% Cl)** | **P-value** | **Unadjusted**  **OR (95% Cl)** | **P-value** | **Unadjusted**  **OR (95% Cl)** | **P-value** | **Unadjusted**  **OR (95% Cl)** | **P-value** |
|  |  |  |  |  |  |  |  |  |  |  |  |  |  |  |
| **Ethnicity** |  |  |  |  |  |  |  |  |  |  |  |  |  |  |
| **White** | Ref |  | Ref |  | Ref |  | Ref |  | Ref |  | Ref |  | Ref |  |
| **Asian** | 1.32 (0.90 - 1.92) | 0.154 | 0.61 (0.24 - 1.54) | 0.294 | 0.97 (0.62 - 1.52) | 0.889 | 0.84 (0.57 - 1.23) | 0.378 | 1.35 (0.92 - 1.99) | 0.128 | 1.04 (0.52 - 2.09) | 0.904 | 1.19 (0.81 - 1.73) | 0.377 |
| **Black** | 1.23 (0.55 - 2.75) | 0.608 | - | - | 0.84 (0.31 - 2.26) | 0.728 | 0.52 (0.23 - 1.15) | 0.105 | 0.68 (0.27 - 1.73) | 0.424 | 1.04 (0.24 - 4.52) | 0.955 | 1.55 (0.68 - 3.55) | 0.298 |
| **Mixed** | 1.26 (0.64 - 2.46) | 0.507 | 1.82 (0.62 - 5.33) | 0.275 | 1.48 (0.71 - 3.05) | 0.293 | 1.12 (0.55 - 2.27) | 0.750 | 1.08 (0.53 - 2.20) | 0.824 | 1.94 (0.73 - 5.14) | 0.185 | 1.54 (0.77 - 3.09) | 0.219 |
| **Other** | 1.05 (0.29 - 3.73) | 0.945 | - | - | 0.84 (0.18 - 3.98) | 0.825 | 1.31 (0.34 - 5.10) | 0.698 | 0.54 (0.11 - 2.57) | 0.440 | - | - | 0.58 (0.16 - 2.08) | 0.404 |

**SUPPLEMENTARY MATERIAL 6.** Sensitivity analysis of factors associated with long COVID and its clusters of symptoms preceding imputation of the data

| **Variable** | **Total N=1,067** | | | | | | | | | | | | | |  |
| --- | --- | --- | --- | --- | --- | --- | --- | --- | --- | --- | --- | --- | --- | --- | --- |
|  |  |  |  |  |  |  |  |  |  |  |  |  |  |  |  |
|  |  |  |  |  |  |  |  |  |  |  |  |  |  |  |  |
|  | **Cardiopulmonary** | | **Gastrointestinal** | | **Musculoskeletal** | | **Neurocognitive & Neurologic** | | **Psychological & Social** | | **Upper Respiratory Tract** | | **Systemic** | |  |
|  | **Unadjusted OR (95% Cl)** | **P-value** | **Unadjusted OR (95% Cl)** | **P-value** | **Unadjusted OR (95% Cl)** | **P-value** | **Unadjusted OR (95% Cl)** | **P-value** | **Unadjusted OR (95% Cl)** | **P-value** | **Unadjusted OR (95% Cl)** | **P-value** | **Unadjusted OR (95% Cl)** | **P-value** |  |
|  |  |  |  |  |  |  |  |  |  |  |  |  |  |  |  |
| **Ethnicity** |  |  |  |  |  |  |  |  |  |  |  |  |  |  |  |
| **White** | Ref |  | Ref |  | Ref |  | Ref |  | Ref |  | Ref |  | Ref |  |  |
| **Asian** | 1.32 (0.90 - 1.92) | 0.154 | 0.61 (0.24 - 1.54) | 0.294 | 0.97 (0.62 - 1.52) | 0.889 | 0.84 (0.57 - 1.23) | 0.378 | 1.35 (0.92 - 1.99) | 0.128 | 1.04 (0.52 - 2.09) | 0.904 | 1.19 (0.81 - 1.73) | 0.377 |  |
| **Black** | 1.23 (0.55 - 2.75) | 0.608 |  |  | 0.84 (0.31 - 2.26) | 0.728 | 0.52 (0.23 - 1.15) | 0.105 | 0.68 (0.27 - 1.73) | 0.424 | 1.04 (0.24 - 4.52) | 0.955 | 1.55 (0.68 - 3.55) | 0.298 |  |
| **Mixed** | 1.26 (0.64 - 2.46) | 0.507 | 1.82 (0.62 - 5.33) | 0.275 | 1.48 (0.71 - 3.05) | 0.293 | 1.12 (0.55 - 2.27) | 0.750 | 1.08 (0.53 - 2.20) | 0.824 | 1.94 (0.73 - 5.14) | 0.185 | 1.54 (0.77 - 3.09) | 0.219 |  |
| **Other** | 1.05 (0.29 - 3.73) | 0.945 |  |  | 0.84 (0.18 - 3.98) | 0.825 | 1.31 (0.34 - 5.10) | 0.698 | 0.54 (0.11 - 2.57) | 0.440 |  |  | 0.58 (0.16 - 2.08) | 0.404 |  |

|  | **Cardiopulmonary** | | **Gastrointestinal** | | **Musculoskeletal** | | **Neurologic** | | **Psychological and social** | | **URT** | | **Systemic** | | **Other** | |  |
| --- | --- | --- | --- | --- | --- | --- | --- | --- | --- | --- | --- | --- | --- | --- | --- | --- | --- |
|  | **adjusted OR** | **P-value** | **adjusted OR** | **P-value** | **adjusted OR** | **P-value** | **adjusted OR** | **P-value** | **adjusted OR** | **P-value** | **adjusted OR** | **P-value** | **adjusted OR** | **P-value** | **adjusted OR** | **P-value** |  |
|  |  |  |  |  |  |  |  |  |  |  |  |  |  |  |  |  |  |
| **Ethnicity** |  |  |  |  |  |  |  |  |  |  |  |  |  |  |  |  |  |
| **White** | Ref |  | Ref |  | Ref |  | Ref |  | Ref |  | Ref |  | Ref |  | Ref |  |  |
| **Asian** | 1.25 (0.83 - 1.90) | 0.286 | 0.46 (0.14 - 1.51) | 0.200 | 1.14 (0.70 - 1.86) | 0.587 | 0.87 (0.57 - 1.33) | 0.532 | 1.39 (0.91 - 2.13) | 0.132 | 0.96 (0.42 - 2.18) | 0.928 | 1.11 (0.74 - 1.69) | 0.609 | 1.20 (0.59 - 2.44) | 0.609 |  |
| **Black** | 1.42 (0.59 - 3.38) | 0.433 |  |  | 1.05 (0.38 - 2.91) | 0.933 | 0.53 (0.22 - 1.26) | 0.151 | 0.53 (0.18 - 1.60) | 0.262 | 1.29 (0.29 - 5.69) | 0.737 | 1.75 (0.70 - 4.39) | 0.233 | 1.14 (0.26 - 5.03) | 0.861 |  |
| **Mixed** | 1.12 (0.54 - 2.32) | 0.761 | 0.95 (0.22 - 4.09) | 0.941 | 1.16 (0.51 - 2.65) | 0.723 | 1.02 (0.48 - 2.16) | 0.961 | 1.04 (0.48 - 2.25) | 0.917 | 2.40 (0.89 - 6.50) | 0.085 | 1.35 (0.65 - 2.82) | 0.426 | 1.54 (0.52 - 4.53) | 0.436 |  |
| **Other** | 1.20 (0.27 - 5.44) | 0.809 |  |  | 1.45 (0.27 - 7.64) | 0.663 | 1.61 (0.31 - 8.44) | 0.575 |  |  |  |  | 0.35 (0.07 - 1.83) | 0.213 | 2.04 (0.24 - 17.56) | 0.515 |  |
| **Age*** | 1.05 (0.94 - 1.18) | 0.402 | 1.00 (0.79 - 1.27) | 0.989 | 1.15 (1.01 - 1.32) | 0.042 | 1.04 (0.92 - 1.17) | 0.516 | 0.97 (0.86 - 1.10) | 0.654 | 1.18 (0.95 - 1.47) | 0.138 | 0.92 (0.82 - 1.03) | 0.137 | 1.05 (0.86 - 1.29) | 0.602 |  |
| **Sex** |  |  |  |  |  |  |  |  |  |  |  |  |  |  |  |  |  |
| Male | Ref |  |  | Ref | Ref |  | Ref |  | Ref |  | Ref |  | Ref |  | Ref |  |  |
| Female | 1.22 (0.86 - 1.72) | 0.271 | 3.87 (1.19 - 12.60) | 0.025 | 1.64 (1.07 - 2.53) | 0.025 | 1.63 (1.16 - 2.29) | 0.005 | 1.28 (0.88 - 1.87) | 0.196 | 1.47 (0.73 - 2.95) | 0.278 | 0.98 (0.69 - 1.37) | 0.886 | 2.03 (0.99 - 4.17) | 0.054 |  |

|  | **Cardiopulmonary** | | | | **Gastrointestinal** | | | | **Musculoskeletal** | | | | **Neurologic** | | | | **Psychological and social** | | | | **URT** | | | | **Systemic** | | | | **Other** | | | |
| --- | --- | --- | --- | --- | --- | --- | --- | --- | --- | --- | --- | --- | --- | --- | --- | --- | --- | --- | --- | --- | --- | --- | --- | --- | --- | --- | --- | --- | --- | --- | --- | --- |
|  | **adjusted OR** |  |  | **P-value** | **adjusted OR** |  |  | **P-value** | **adjusted OR** |  |  | **P-value** | **adjusted OR** |  |  | **P-value** | **adjusted OR** |  |  | **P-value** | **adjusted OR** |  |  | **P-value** | **adjusted OR** |  |  | **P-value** | **adjusted OR** |  |  | **P-value** |
|  |  |  |  |  |  |  |  |  |  |  |  |  |  |  |  |  |  |  |  |  |  |  |  |  |  |  |  |  |  |  |  |  |
| **Ethnicity** |  |  |  |  |  |  |  |  |  |  |  |  |  |  |  |  |  |  |  |  |  |  |  |  |  |  |  |  |  |  |  |  |
| **White** | Ref |  |  |  | Ref |  |  |  | Ref |  |  |  | Ref |  |  |  | Ref |  |  |  | Ref |  |  |  | Ref |  |  |  | Ref |  |  |  |
| **Asian** | 1.503308 | 0.900737 | 2.508984 | 0.119 | 0.480553 | 0.131576 | 1.755109 | 0.268 | 1.177141 | 0.636797 | 2.175987 | 0.603 | 0.869487 | 0.517509 | 1.460862 | 0.597 | 1.447712 | 0.852683 | 2.457971 | 0.171 | 0.988714 | 0.388485 | 2.516329 | 0.981 | 1.263526 | 0.756767 | 2.109631 | 0.371 | 1.441415 | 0.603908 | 3.440384 | 0.410 |
| **Black** | 1.828456 | 0.675008 | 4.952903 | 0.235 | 1 |  |  |  | 1.200721 | 0.401017 | 3.595183 | 0.744 | 0.424972 | 0.15759 | 1.146023 | 0.091 | 0.633778 | 0.199629 | 2.012099 | 0.439 | 0.519944 | 0.064903 | 4.165295 | 0.538 | 1.488642 | 0.528817 | 4.190589 | 0.451 | 1.531335 | 0.328364 | 7.141434 | 0.588 |
| **Mixed** | 0.911226 | 0.390412 | 2.126814 | 0.830 | 1.10265 | 0.24442 | 4.974368 | 0.899 | 0.660215 | 0.218244 | 1.997226 | 0.462 | 0.694935 | 0.304721 | 1.584844 | 0.387 | 1.237859 | 0.529075 | 2.89618 | 0.623 | 1.718188 | 0.481018 | 6.137345 | 0.405 | 1.409756 | 0.610899 | 3.253258 | 0.421 | 1.985375 | 0.642732 | 6.132752 | 0.233 |
| **Other** | 1.444344 | 0.30527 | 6.833723 | 0.643 | 1 |  |  |  | 1.350796 | 0.234198 | 7.791068 | 0.737 | 1.403672 | 0.254575 | 7.739552 | 0.697 | 1 |  |  |  | 1 |  |  |  | 0.397263 | 0.072371 | 2.18067 | 0.288 | 3.604955 | 0.366851 | 35.42498 | 0.271 |
| **Migration Status** |  |  |  |  |  |  |  |  |  |  |  |  |  |  |  |  |  |  |  |  |  |  |  |  |  |  |  |  |  |  |  |  |
| **Born in UK** | Ref |  |  |  | Ref |  |  |  | Ref |  |  |  | Ref |  |  |  | Ref |  |  |  | Ref |  |  |  | Ref |  |  |  | Ref |  |  |  |
| **Born abroad** | 0.758125 | 0.500605 | 1.148119 | 0.191 | 1.426846 | 0.637651 | 3.192797 | 0.387 | 1.213147 | 0.754482 | 1.950644 | 0.425 | 1.27229 | 0.832953 | 1.943355 | 0.265 | 1.051941 | 0.685146 | 1.615101 | 0.817 | 2.011265 | 1.027399 | 3.937307 | 0.041 | 1.056777 | 0.702372 | 1.590009 | 0.791 | 0.497299 | 0.221859 | 1.114701 | 0.090 |
| **Age, per decade increase** | 1.011936 | 0.887305 | 1.154073 | 0.860 | 1.053853 | 0.81506 | 1.362606 | 0.689 | 1.147308 | 0.981625 | 1.340956 | 0.084 | 1.003492 | 0.87795 | 1.146985 | 0.959 | 0.937249 | 0.816318 | 1.076095 | 0.358 | 1.137353 | 0.889116 | 1.454896 | 0.306 | 0.884666 | 0.776146 | 1.008359 | 0.066 | 0.972038 | 0.773408 | 1.221682 | 0.808 |
| **Sex** |  |  |  |  |  |  |  |  |  |  |  |  |  |  |  |  |  |  |  |  |  |  |  |  |  |  |  |  |  |  |  |  |
| **Male** | Ref |  |  |  | Ref |  |  |  | Ref |  |  |  | Ref |  |  |  | Ref |  |  |  | Ref |  |  |  | Ref |  |  |  | Ref |  |  |  |
| **Female** | 1.199178 | 0.803184 | 1.790408 | 0.374 | 3.526813 | 1.052259 | 11.82068 | 0.041 | 1.558372 | 0.940972 | 2.580867 | 0.085 | 1.64746 | 1.111761 | 2.441283 | 0.013 | 1.063764 | 0.692819 | 1.63332 | 0.778 | 1.272618 | 0.586157 | 2.763009 | 0.542 | 0.953247 | 0.644108 | 1.410758 | 0.811 | 1.538094 | 0.716221 | 3.303074 | 0.270 |
| **No. of COVID vaccines at time of acute infection** |  |  |  |  |  |  |  |  |  |  |  |  |  |  |  |  |  |  |  |  |  |  |  |  |  |  |  |  |  |  |  |  |
| **0** | Ref |  |  |  | Ref |  |  |  | Ref |  |  |  | Ref |  |  |  | Ref |  |  |  | Ref |  |  |  | Ref |  |  |  | Ref |  |  |  |
| **1** | 0.698767 | 0.368271 | 1.32586 | 0.273 | 1.185538 | 0.340431 | 4.128592 | 0.789 | 0.531731 | 0.229168 | 1.233755 | 0.141 | 0.620663 | 0.333156 | 1.156284 | 0.133 | 0.635485 | 0.310925 | 1.298838 | 0.214 | 1.086355 | 0.362127 | 3.258984 | 0.883 | 0.61718 | 0.332045 | 1.147167 | 0.127 | 1 |  |  |  |
| **2** | 0.496249 | 0.28257 | 0.871511 | 0.015 | 1.132868 | 0.421411 | 3.045459 | 0.805 | 0.429631 | 0.205905 | 0.896445 | 0.024 | 0.564998 | 0.336046 | 0.949939 | 0.031 | 1.258275 | 0.736249 | 2.150435 | 0.401 | 0.737824 | 0.25383 | 2.144683 | 0.577 | 0.577865 | 0.343086 | 0.973308 | 0.039 | 0.496863 | 0.172795 | 1.428708 | 0.194 |
| **3** | 0.713931 | 0.489165 | 1.041973 | 0.081 | 1.070392 | 0.520745 | 2.200191 | 0.853 | 0.53733 | 0.335304 | 0.861081 | 0.010 | 0.675532 | 0.464275 | 0.982917 | 0.040 | 1.049747 | 0.710541 | 1.550887 | 0.807 | 0.691841 | 0.326104 | 1.467766 | 0.337 | 0.912297 | 0.630185 | 1.320701 | 0.627 | 0.651869 | 0.32966 | 1.289004 | 0.219 |
| **Occupation** |  |  |  |  |  |  |  |  |  |  |  |  |  |  |  |  |  |  |  |  |  |  |  |  |  |  |  |  |  |  |  |  |
| **Doctor or medical support** | Ref |  |  |  | Ref |  |  |  | Ref |  |  |  | Ref |  |  |  | Ref |  |  |  | Ref |  |  |  | Ref |  |  |  | Ref |  |  |  |
| **Nurse, NA or Midwife** | 1.445734 | 0.892601 | 2.341635 | 0.134 | 1.497439 | 0.462127 | 4.852186 | 0.501 | 2.214668 | 1.197335 | 4.096393 | 0.011 | 1.179284 | 0.72848 | 1.909059 | 0.502 | 2.02734 | 1.199544 | 3.426392 | 0.008 | 2.102166 | 0.758669 | 5.824811 | 0.153 | 2.016511 | 1.250571 | 3.251568 | 0.004 | 0.96752 | 0.422164 | 2.217374 | 0.938 |
| **Allied Health Professional**** | 1.116212 | 0.715001 | 1.742556 | 0.629 | 1.75515 | 0.570248 | 5.402128 | 0.327 | 1.924785 | 1.074037 | 3.449413 | 0.028 | 1.429425 | 0.918317 | 2.225002 | 0.114 | 1.601396 | 0.982035 | 2.611383 | 0.059 | 2.443354 | 0.942059 | 6.337161 | 0.066 | 1.725226 | 1.114458 | 2.67072 | 0.014 | 1.157317 | 0.54151 | 2.47342 | 0.706 |
| **Dental** | 0.923323 | 0.42051 | 2.027363 | 0.842 | 2.523179 | 0.574146 | 11.08852 | 0.22 | 1.454892 | 0.54132 | 3.910278 | 0.457 | 0.99375 | 0.464149 | 2.127636 | 0.987 | 1.292013 | 0.557838 | 2.992442 | 0.550 | 1.290592 | 0.238399 | 6.986727 | 0.767 | 0.942603 | 0.440296 | 2.017961 | 0.879 | 0.556127 | 0.112841 | 2.740832 | 0.471 |
| **Admin, estates or other** | 1.022809 | 0.516947 | 2.023683 | 0.948 | 2.900743 | 0.751981 | 11.18953 | 0.122 | 1.816772 | 0.781396 | 4.224055 | 0.165 | 0.90535 | 0.465804 | 1.759662 | 0.769 | 0.866466 | 0.397312 | 1.88961 | 0.719 | 0.961456 | 0.18023 | 5.128982 | 0.963 | 1.039398 | 0.536354 | 2.014246 | 0.909 | 0.583238 | 0.15018 | 2.265054 | 0.436 |
